# Intersections among EMA and smartwatch‐derived daily sleep, physical activity, stress, mood, and subjective cognition in older adults without dementia

**DOI:** 10.1002/alz70857_107237

**Published:** 2025-12-26

**Authors:** Riya Chaturvedi, Sophia L. Holmqvist, Marina Kaplan, Tania Giovanetti

**Affiliations:** ^1^ Temple University, Philadelphia, PA, USA

## Abstract

**Background:**

It is well known that greater intraindividual variability (IIV) in sleep and greater daily stress are associated with worse subjective cognition. It has been less explored how IIV in mood as well as daily physical activity may be related to day‐to‐day IIV in subjective cognition using EMA and smartwatches concurrently. This study sought to investigate what modifiable risk factors (physical activity, sleep, stress, and mood) captured through EMA and a smartwatch relate to day‐to‐day fluctuations in subjective cognition as well as concordance of smartwatch and EMA measured physical activity and sleep duration.

**Method:**

Fifty‐five community dwelling older adults with healthy cognition (*n* = 45; 30.9% Black/African American) or mild cognitive impairment (MCI; n = 10) wore a Garmin Vivosmart4 smartwatch 23 hours/day and completed a 9‐question daily EMA smartphone‐based survey for 30 days. EMA questions included self‐reported mood (happy, neutral, sad) subjective cognition (not sharp, neutral, very sharp), stress (1‐10), exercise time, and sleep duration. Daily step count and sleep duration were captured from the smartwatch. Individual‐level averages were calculated for all EMA and SW variables. Intraindividual standard deviations were calculated for mood, subjective cognition, and sleep duration. Spearman correlations were run to examine relations among EMA and smartwatch variables.

**Result:**

Greater IIV EMA sleep duration was related to greater IIV smartwatch sleep duration (ρ = .276, *p* = .041) and greater IIV subjective cognition (ρ = .272, *p* = .045). Greater stress was related to IIV subjective cognition (ρ = .388, *p* = .003). Greater self‐reported EMA exercise was related to greater daily step count (ρ = .443, *p* < .001) and lower IIV subjective cognition (ρ = ‐.281, *p* = .037). Greater IIV mood was related to greater IIV subjective cognition (ρ = .336, *p* = .012).

**Conclusion:**

Inconsistent sleep patterns, greater daily stress, less physical activity, and more variable mood were related to increased day‐to‐day fluctuations in subjective daily cognition. There was concordance of EMA vs. smartwatch derived metrics for physical activity and sleep. Only EMA and not smartwatch measures of IIV sleep duration and average physical activity were related to fluctuations in subjective cognition. Overall, these findings emphasize the importance of regularity in daily mood and sleep as well as increasing daily exercise as modifiable risk factors for cognitive decline.

